# Ketamine in fibromyalgia: a systematic review

**DOI:** 10.1186/s42358-024-00393-9

**Published:** 2024-07-29

**Authors:** Jozélio Freire de Carvalho, Eduardo Pondé de Sena

**Affiliations:** Instituto de Ciencias da Saude da Universidade Federal da Bahia, Rua das Violetas, 42, ap. 502, Pituba, Salvador, Bahia Brazil

**Keywords:** Ketamine, Fibromyalgia, Chronic pain, Anesthetics, Depression

## Abstract

**Objective:**

Fibromyalgia (FM) subjects are treated with antidepressant agents; in most cases, these drugs lose efficacy or have adverse effects. Ketamine is an anesthetic drug used in FM in some studies. This article aims to systematically review the safety and efficacy of ketamine in fibromyalgia (FM) patients.

**Materials and methods:**

We systematically searched articles on FM and ketamine published at Pubmed from 1966 to 2021. This study was registered at PROSPERO.

**Results:**

There were only 6 articles published in this field, with a total of 115 patients. The female sex was predominant (88 to 100%). The age varied from 23 to 53 years old. Disease duration ranged from 1 month to 28 years. The dosage of ketamine changed from 0.1 mg/kg-0.3-0.5 mg/kg in intravenous infusion (4/5) and subcutaneous application (1/5). Regarding outcomes, the Visual analog scale (VAS) before ketamine was from 59 to 100 mm and after treatment from 2 to 95 mm. Most short-term studies had a good response. Only the study with 8 weeks of follow-up did not observe a good response. Side effects were common; all appeared during the infusion and disappeared after a few minutes of the ketamine injection.

**Conclusions:**

The present study demonstrates the effectiveness and safety of ketamine in FM patients in the short term. Although, more studies, including long-term follow-up studies, are still needed.

## Key summary bullet points

• Ketamine is an anesthetic drug used in fibromyalgia (FM) in some trials.

• We systematically searched articles on FM and ketamine and found 6 articles with 115 patients.

• Ketamine reduced pain Visual analog scale (VAS) parameters with mild side effects.

## Introduction

Fibromyalgia (FM) is a chronic disease characterized by diffuse pain over the body, with associated comorbidities, including anxiety, depression, cephalalgia, irritable bowel syndrome, and other manifestations [1]. FM is the third most common musculoskeletal condition and may affect from 0.4 to 8.8% of the global population [1].

The classical treatment of FM includes physical exercise, psychological interventions, and drugs. Regarding pharmacological treatment, the use of antidepressants is the primary option for this condition. Though side effects may lead to dropouts, the rate varies from 9 to 27.2% in the published articles [2]. A Cochrane review [3] observed insufficient evidence to support or disprove drug therapy combinations’ routine use for managing fibromyalgia pain. Pragmatic clinical trials are needed [4], considering differences between individuals related to drug response and investigating predictors of successful management of this chronic pain syndrome [5]. The absence of efficacy is also seen during FM therapy, which may reach 50 to 60% of the cases [1]. In this manner, novel therapeutic modalities are desired for refractory patients or those with adverse drug effects.

Ketamine is an anesthetic drug and noncompetitive antagonist of the N-methyl-D-aspartate receptor that induces dissociative (psychedelic) effects and is used for depression therapy and pain conditions, such as refractory headaches [6, 7]. There are some studies of ketamine use in FM patients, although a systematic review has not been performed in this field until now.

This article aims to perform a systematic review of ketamine in FM patients.

## Methods

### Literature review

We have systematically studied articles published in PubMed, Web of Sciences, LILACS, and SciELO from 1966 to November 2021 using the following MeSH terms: “ketamine” and “fibromyalgia.” We used equivalent strategies in the other databases. All the related articles are based on “ketamine” and “fibromyalgia” without language restriction. The reference lists of the selected articles were analyzed to identify other publications. Initially, two independent investigators (JFC and EDS) performed the literature search and independently selected the study abstracts. In the second stage, the same reviewers independently read the full-text articles selected by abstracts. The authors followed the PRISMA guidelines [8]. We designed a standardized form to extract the following information from relevant articles regarding the authors and year of publication, the number of patients studied, demographic data, disease duration, study follow-up, Visual analog scale (VAS) pre- and post-interventions, ketamine posology, and outcomes. The primary outcome was the reduction of VAS. And secondary outcome was the frequency of side effects.

This study was registered with the Prospective Register of Systematic Reviews (PROSPERO) under CRD42020221380.

The Cochrane Collaboration Risk of Bias Tool was used to evaluate bias risk [9].

The search strategy used was: “ketamine” AND “fibromyalgia. “Unfortunately, the authors did not perform a meta-analysis.

## Results

**Fig. 1:**
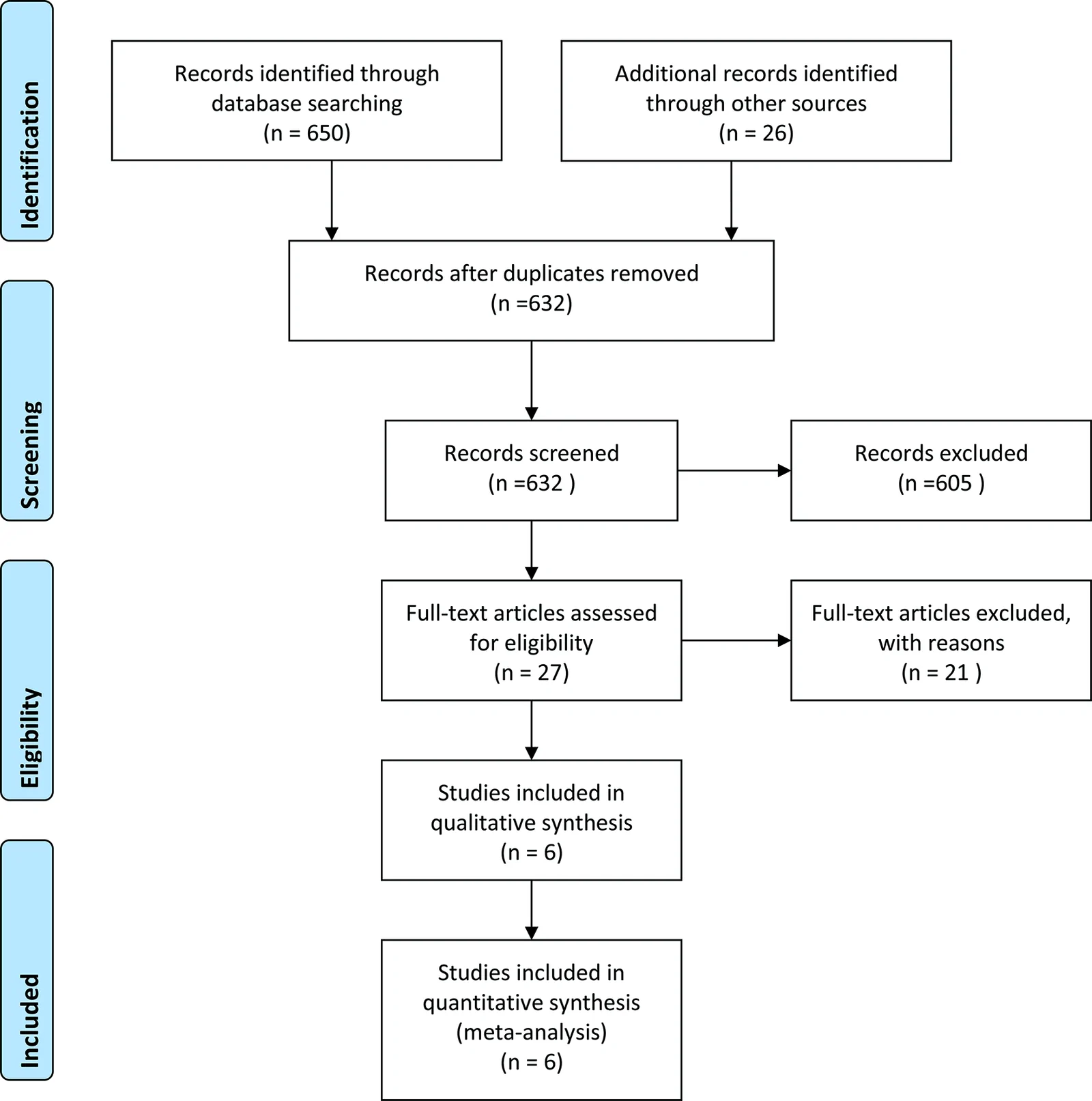
Flow chart of the included articles, following PRISMA

Figure 1 shows the flowchart of the included articles.

The demographics, clinical, and VAS pre- and post-ketamine treatment features of the patients with FM are shown in Table 1 [10–15].

**Table 1:** Clinical and demographic characteristics of the six studies on FM and ketamine

Author, year	Study design	*N*, female sex	Age, years	Disease duration	Follow-up study	Ketamine prescription	VAS, pre and post, ketamine	VAS, pre and post, placebo	Side effects
Noppers et al. [10]	RCT, prospective, double-blind	24, 96%	42.1 ± 11	16 (1–192) months	8 weeks	0.5 mg/kg IV in 30 min or midazolam	54 ± 6 → 31 ± 8 at 180 min	58 ± 4 → 43 ± 7 at 180 min	Minutes after, drowsy and felt high in ketamine group
Guedj et al. [13]	Prospective	17, 100%	48.5 ± 11	NA	2 weeks	100 mg/day for 10 days SC	81.8 ± 4.2 → 31.8 ± 27.1 at 2 weeks	No	NA
Cohen et al. [15]	Open prospective	34, 88%	44.2	NA	NA	0.1 mg/kg IV in 7 min	58 ± 19 → 30 ± 24, at in the end of infusion	58 ± 19 → 56 ± 19 in the end of infusion	24 (71%)-dizziness, euphoria, confusion, nausea
Muller et al. [11]	Double-blind, cross-over placebo-controlled	20, 100%	42.05 ± 4.36	N/A	3 days and 3 months	1 mg/kg/day (IV continuous infusion for 3 days)	63.8 → 45.5 after 3 days of infusions and 63.8 → 56.0 after the second hospitalization after termination of 3-day infusions after 3 months	62.2 → 51.2 5 after 3 days of infusions and 62.2 → 67.5 after the second hospitalization after termination of 3-day infusions after 3 months	Superficial phlebitis. The other effects were similar to placebo
Graven-Nielsen, et al. [14]	RCT, double-blind, cross-over	29, 100%	45	3.2 years	150 min each session	0.3 mg/kg IV in 30 min vs. placebo on 2 separated sessions	~ 42 → 12 at 30 min	~ 36 → 36 at 30 min	NA
Sörensen et al. [12]	RCT, prospective, double-blind	11, 100%	39 (23–53)	3 (3–28) years	30 min and 1 week	0.3 mg/kg IV in 10 min vs. placebo	58 (2–100) → 53 (2–95) at 1 week after	46 (2–93) → 50 (2–100) at 1 week after	10 (91%)–unreality feeling (*n* = 5), dizziness (*n* = 4), hearing changes (*n* = 3). All disappeared after 15 min of infusion.

*IV* intravenous, *NA* not available, *RCT* randomized controlled trial, *SC* subcutaneous, *VAS* visual analogic scale

There were only 6 articles published in this field, with a total of 115 patients. The female sex was predominant and varied from 88 to 100% of the studies. The ages varied from 23 to 53 years old. Disease duration ranged from 1 month to 28 years.

The dosage of ketamine varied from 0.1 mg/kg as a low dose to 0.3–0.5 mg/kg, usually in intravenous infusion (4/5) and subcutaneous application in only one study. The follow-up period was from minutes to 8 weeks.

**Fig. 2:**
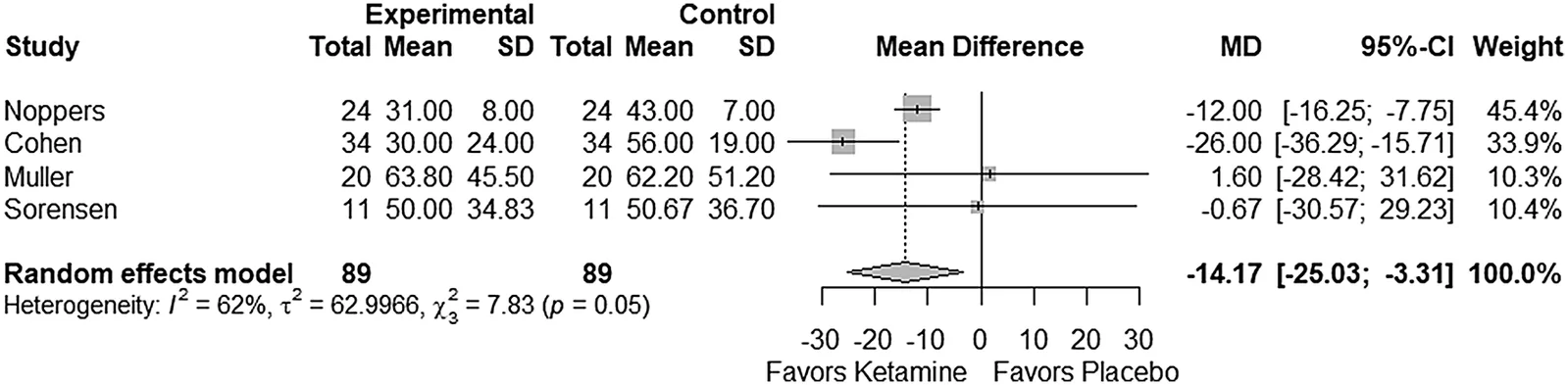
Forest plot of the included studies

Regarding outcomes, most studies evaluated the VAS, and before ketamine, VAS was from 52 to 100 mm, and after treatment, from 2 to 95 mm. Although short-term studies had a good response, only the study with 8 weeks of follow-up did not observe a good response. Figure 2 shows forest plot of the included studies.

Side effects were common and varied from 71 to 91% of the patients. However, most of them were mild or moderate and comprised: of unreality feelings), dizziness), and hearing changes). Furthermore, all appeared during infusion and disappeared after a few minutes of the ketamine injection.

**Fig. 3:**
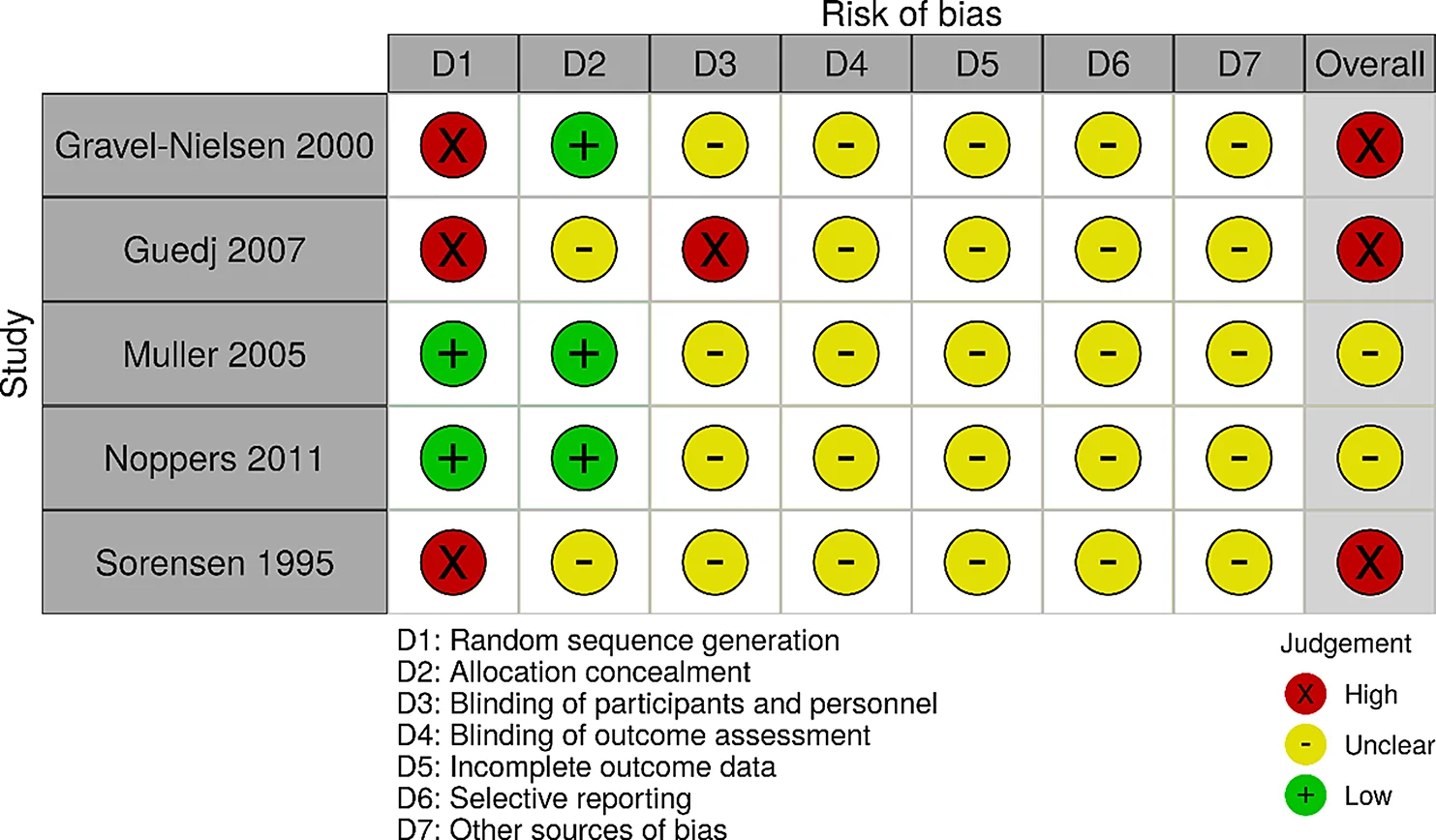
Table presenting the risk of bias from 5 randomized studies

The bias risk was evaluated (Fig. 3) and showed that 3 studies had a high risk of bias, and in the other 2 articles, this risk was unclear. We did not see heterogeneity in the included studies.

## Discussion

This article systematically reviewed for the first time the therapeutical effects of ketamine in FM patients.

The strengths of this study included the inclusion of studies with patients that fulfilled the international criteria for FM; and, second, the exclusion of case reports, case series, and observational studies. In this line, prospective studies are those with higher evidence degrees.

Ketamine was introduced as a dissociative anesthetic in 1965 and displayed analgesic, anesthetic, psychosensory, anti-inflammatory, and antidepressant properties [16]. Its primary action mechanism includes the noncompetitive blockade of N-Methyl-D-Aspartate (NMDA) receptors in the anterior cingulate, insula, prefrontal cortex, and dorsal horn neurons of the spinal cord. This blockage reduces calcium influx with a consequent decrease in glutamate receptor activity [16]. Furthermore, ketamine also acts on an opioid system via opioid receptors; in the noradrenergic, serotoninergic, and dopaminergic systems and the muscarinic and nicotinic acetylcholine receptors [16].

A recent meta-analysis explored the effectiveness of randomized controlled trials of intravenous ketamine compared to a placebo for pain relief in chronic diseases. The authors aimed to examine the lowest recorded pain score ≥ 48 h after treatment interruption, and secondary outcomes comprised response rates and tolerability. Seven studies with 211 patients investigating neuropathic (*n* = 2), non-neuropathic (nociplastic or nociceptive) (*n* = 3), and mixed pain (*n* = 2) were included. Small sample sizes (median sample size of 24 were a study limitation. After the infusion and up to 14 days of ketamine, minor effects were significantly seen. However, for the three studies reporting response rates, a positive outcome was more significant in the ketamine than in the placebo group [17].

A study in the Netherlands [8] examined the analgesic effect of esketamine in a randomized, double-blind trial controlled by midazolam in 24 FM patients. Research volunteers were randomized to receive an intravenous infusion with either esketamine or midazolam. Visual analog scale (VAS) scores and pain scores derived from the fibromyalgia impact questionnaire (FIQ) were obtained minutes after the end of the infusion and weekly during an 8-week follow-up. After 15 min, a significant reduction in pain scores > 50%, compared to midazolam, was observed. Although, for the VAS and FIQ scores, no significant differences were observed in the treatment effects within 2.5 h after infusion or during the 8-week follow-up. Side effects assessed by the Bowdle questionnaire were mild to moderate in both study groups and decreased rapidly. The effectiveness of esketamine was limited by its pharmacokinetics. The authors concluded that a short-term infusion of esketamine is unsatisfactory at inducing long-term analgesia in FM patients.

A French study [11] evaluated the recovery of pain from hyperalgesic fibromyalgia with single-photon tomography of ethyl perfusion ethyl cysteine (SPECT) after administration of ketamine in a group of 17 women, 11 of whom were considered “good responders,” with decreased pain intensity, assessed by the VAS. In contrast, six volunteers were considered “bad responders.” The authors found distinct brain functional SPECT patterns between those who responded and those who did not. Additionally, the difference in cerebral blood flow in the midbrain after ketamine injection was positively associated with reduced VAS pain scores.

A study in the United States of America [13] evaluated whether a ketamine test could predict the response to a therapeutic trial with oral dextromethorphan (DX) in 34 patients with fibromyalgia. The cut-off value for a positive response to the ketamine test was 67% pain relief, and a positive reaction to DX treatment was a 50% reduction in pain at 4-to-6-week follow-up visits. In addition, the correlation between pain relief with ketamine and DX was highly significant (*P* < 0.001). In the end, 10 patients responded positively to ketamine and DX, 19 responded to no drugs, 3 responded positively to ketamine but not DX, and 2 achieved adequate pain relief with DX but not ketamine.

Another article from France investigated the effects of ketamine infusions for three days on fibromyalgia pain [9]. This double-blind crossover study was intended to evaluate pain relief on the VAS. Patients were hospitalized for three-day ketamine vs. placebo infusions. As a crossover design, patients allocated to ketamine were then submitted to placebo (and vice-versa) in another 3-day hospitalization after 3 months. The ketamine infusion at a rate of 1 mg/kg/day for three days showed no significant effect on spontaneous pain if we consider all the patients. However, a subgroup of patients insensitive to placebo had 30% pain relief for at least two months.

A study in Sweden assessed the effects of a placebo or ketamine given over 30 min on two separate occasions in FM patients [14]. Pain intensity was evaluated employing the VAS. At first, 29 FMS patients received ketamine or isotonic saline to determine which subjects were ketamine responders (50% decrease in pain intensity at rest by the active drug on two consecutive VAS assessments). The authors found a pain intensity reduction in the ketamine group, and local and referred pain areas were also reduced in this group [14].

Another Swedish study [12] examined pain intensity and tolerance, pain pressure threshold, muscle strength, and static muscle endurance in 31 FM subjects before and after intravenous ketamine (11 patients), morphine (9 patients), and lidocaine (11 patients) injections. The ketamine group showed decreased pain intensity and tenderness at tender points [12].

In contrast to the lack of randomized controlled trials, systematic reviews, and meta-analyses of ketamine in fibromyalgia, there are many publications on mood disorders, particularly about its effectiveness in resistant depression and the management of suicidal ideation [17–20]. The first studies already pointed to a potent reduction in depressive symptoms in single doses [18]. Despite the encouraging results, strategies for maintaining improvement are the target of intense research. Repeated infusions of ketamine in subanesthetic doses (<0.5–2 mg/kg) are helpful to prolong the administration effect in the short term [21]. Several studies have been carried out in recent years, including investigations of biomarkers [22], which have multiplied in the literature, and various routes of administration in addition to intravenous (e.g., oral, subcutaneous, intramuscular, and intranasal). A study with the esketamine enantiomer has shown that it is possible, with lower doses, to mitigate dissociative effects [23]. Nasal esketamine has recently been approved in the US and Europe for resistant depression [17].

Finally, a few limitations were seen in the included studies, including no comparison between ketamine with the classical antidepressants used for FM, the number of participants being still low, and the relatively small follow-up. Therefore, future studies should involve large patient samples with more long-term follow-ups, enabling a better understanding of the course of this therapeutic modality in FM.

## Conclusion

This study was a systematic review of all prospective studies that evaluated ketamine and FM patients. Ketamine infusions might be a reasonable therapeutic approach for short-term relief of symptoms but unsatisfactory at inducing long-term analgesia in FM patients. Although, the bias risk is increased in most studies or uncertain in others. Future studies that evaluate the safety and effectiveness of ketamine in FM are desired for long-term follow-up. In patients refractory to conventional therapy, ketamine infusions might be a reasonable therapeutic approach.

## Data Availability

All data are available at request.
